# The Influence of Polymer Superplasticizers on Properties of High-Strength Concrete Based on Low-Clinker Slag Portland Cement

**DOI:** 10.3390/ma16052075

**Published:** 2023-03-03

**Authors:** Leonid Dvorkin, Vadim Zhitkovsky, Ruslan Makarenko, Yuri Ribakov

**Affiliations:** 1 Institute of Civil Engineering and Architecture, National University of Water and Environmental Engineering, 33028 Rivne, Ukraine; 2 Department of Civil Engineering, Ariel University, Ariel 40700, Israel

**Keywords:** naphthalene formaldehyde, lignosulfonate, polycarboxylate, Portland cement, concrete, compatibility, slag, superplasticizer

## Abstract

The paper deals with the effectiveness of various types of polymers (naphthalene formaldehyde, polycarboxylate, and lignosulfonate) as superplasticizers of concrete mixtures based on low-clinker slag Portland cement. Using the mathematical planning experimental method and statistical models of water demand of concrete mixtures with polymer superplasticizers, as well as concrete strength at different ages and under different curing conditions (normal curing and after steaming) were obtained. According to the models, the superplasticizer’s water-reducing effect and relative change in concrete strength were obtained. The proposed criterion for evaluating the effectiveness and compatibility of superplasticizers with cement takes into account the water-reducing effect of the superplasticizer and the corresponding relative change in concrete strength. The results demonstrate that the use of the investigated superplasticizer types and low-clinker slag Portland cement allows for achieving a significant increase in concrete strength. The effective contents of various polymer types, which allow the achieving of concrete strengths from 50 MPa to 80 Mpa, has been found.

## 1. Introduction

Superplasticizers (SPs) are used in concrete production since the early 1970s. Using them has allowed significant improvement of concrete properties without increasing cement consumption. Superplasticizers enable obtaining cast and self-compacting mixtures with cone slump of 20 cm and 26 cm, respectively, with a moderate water demand as well as producing high-strength concrete based on ordinary Portland cement and aggregate with low permeability, high corrosion resistance, etc. [1,2,3]. Adding superplasticizers is currently a prerequisite for producing high-performance concrete (HPC), reactive powder concrete, etc.

Most superplasticizers are polymeric substances of various structures [4]. As known [1], superplasticizers can be classified according to their composition and effect (Table 1).

Some of the first SPs were additives that belonged to the joint naphthalene–formaldehyde (NF)-type group. At an additive content of 0.5–1% by cement weight, they allow increasing the concrete mixture cone slump from 2–4 cm to 20–22 cm. Under conditions of equal mixtures workability, as a result of a decrease in W/C, the strength of the concrete with NF SP at 28 days is up to 30–40% higher compared to that without additives. At the same time, the concrete density and water resistance significantly increase, and a number of other properties improve [3].

Using P-type SP, based on polyacrylates and polycarboxylates, reduces the concrete mixtures water demand by more than 30% [2]. If concrete mixtures with traditional SP quickly lose their workability and are not sufficiently stable, mixtures with SP of this type remain in a plastic state for 1.5–2 h. The high retention of concrete mixes with type P SP makes them particularly attractive for monolithic construction and for long-term transportation. Like other SP, they are also successfully used in the precast concrete industry for heat and moisture concrete treatments [2].

The cheapest plasticizers are surfactants based on lignosulfonates. Hydrolysis production wastes—technical lignosulfonates (LST) added into concrete mixtures, usually in an amount of 0.15–0.25% by cement weight, are widely used in concrete technology. Adding 0.8–1% of LST, approximately the same liquefaction can be achieved as with conventional SP content; however, the concrete strength decreases by 1.5 times or more [1]. This is a consequence of a corresponding decrease in the cement hydration degree and an increase in the entrained air volume by 2–2.5 times. The stabilizing effect of LST on the cement hardening processes increases with an increase in the content of the so-called reducing substances (RS) represented by sugars or carbohydrates such as xylose, glucose, galactose, etc. [3].

The SP effect is due to a complex of physicochemical processes in the cement paste-additive system [2]. It is defined mainly by:

- adsorption of mono- or polymolecular surfactants on the surface of mainly hydrate neoplasms;
- colloidal–chemical phenomena at the phases border.

The predominant effect of NF, MF, and LST-type SP (Table 1) is the electrostatic repulsion of cement particles due to the fact that adsorption layers of SP molecules increase the zeta potential on the cement particles’ surface. The value of zeta potential, which has a negative sign, depends on the SP adsorption capacity. An increase in the SP adsorption capacity yields an increase in the hydrocarbon chain length and molecular weight. The role of the zeta potential in the P-type SP mechanism is lower (Table 1) and the mutual repulsion of cement particles is ensured by the so-called steric effect. This effect is due to the chains’ shape, as well as the charge-nature on the cement and hydrates’ grain surface [4,5,6,7,8].

Modern cements have different chemical and mineralogical compositions. The practice of recent decades has shown that adding the same SP in concrete mixtures based on different cements or using the same cement with different superplasticizers has a different character [9,10].

Many studies have been carried out to determine the mineralogical and material composition of cement in assessing the effectiveness of using SP [11]. The classification of cements relative to SP is based on the content of C_3_S and C_3_A minerals [12]; however, this classification determines only the water-reducing activity of SP in concrete mixtures based on Portland cement, without taking into account its reactivity in the presence of SP and mineral admixtures. When active mineral admixtures are added to cement, the achieved effect is affected by their pozzolanic activity and grinding fineness.

Granular blast-furnace slags are the most widely used in cement technology as active mineral admixtures. The addition of SP improves the rheological properties of slags, especially if their dispersion is high [12]. With a significant reduction in water demand due to the synergistic action of the organo–mineral additives components, compressed contact conditions are created [13], under which intensive concrete curing occurs, especially at early stage. According to available data [12], highly dispersed mineral additives in combination with strong SP are the most effective.

In research and practice, cement systems, including active mineral additives, like slag, in an amount of 10 to 70% by cement weight, became popular. It was shown in [14] that it is possible to use CEM III/B slag Portland cement containing from 66 to 80% of slag in composition with SP to obtain high-performance concrete. At the same time, at the present stage, concrete based on low-clinker binders, which according to EN 197-1 [15] belong to CEM III/C slag Portland cements, containing up to 95% blast-furnace slag, are of increased technical, economic, and environmental interest. Investigating the effectiveness of such cement in composition with various types of joint SP is an actual problem [16,17].

Based on available research results [18,19,20], it is possible to assess the compatibility of cement and additives, but this requires special equipment that is not always available in industrial enterprises. In addition, the estimation methods proposed in these and some other works are quite time-consuming.

It was proposed to use three categories of cement–additive compatibility factors: chemical and mineral composition of cement; superplasticizer characteristics: molecular weight, molecular chains structure, and branching, polycondensation degree, as well as such technological parameters as the concentration of additives, temperature, sequence of introduction, mixing mode, etc. [21]. This approach is obviously correct, but it is very difficult to take these factors into account. For example, determining the composition of cement requires a lot of time, and it is expensive.

Another way to assess compatibility was proposed in [22,23]. Based on industrial experience, it was proposed to evaluate the compatibility of superplasticizers of various natures and chemical compositions by their ability to maintain the concrete mix properties for a certain time.

In studies directly related to the compatibility of cements and superplasticizers [18,19,21], it was proposed to use the heat release curves nature as a compatibility criterion. This approach is based on the fact that they can be used to evaluate the additives’ effect on elementary acts occurring during cement hydration, such as adsorption, dissolution, and crystallization. However, these studies consider the use of thermokinetic analysis as a comprehensive tool that not only allows assessing the compatibility but also predicting other properties of concrete. In addition, according to the authors themselves, compatibility can be assessed just qualitatively.

In most studies [1], the compatibility and effectiveness of various SP is determined by their water-reducing effect and a possible increase in concrete strength or a decrease in cement consumption at a given strength. In our opinion, a comprehensive criterion for the effectiveness of joint SPs and their compatibility with types of cement used in concrete can be the ratio of the possible increase in strength (∆*f_cm_*) to the value of the water-reducing effect (WRE):

- water-reducing effect (WRE, %):

(1) $$WRE=\frac{W_{0}-W_{SP}}{W_{0}}100\%,$$

where W_0_ and W_SP_ are the concrete mixture water content before and after SP addition, respectively.

- relative change in compressive strength ($∆f_{cm}$, %):

(2) $$∆f_{cm}=\frac{f_{cm1}-f_{cm0}}{f_{cm0}}100\%,$$

where *f_cm_*_0_ and *f_cm_*_1_ are concrete compressive strength without and with SP, respectively.

When SP additives are compatible with the cement, the ratio ∆*f_cm_*/WRE should be more than 1. With an increase in this ratio, the SP efficiency also increases.

A set of experimental-statistical models, allowing to evaluate the effect of SP type and content at various slag Portland cement consumption on concrete mixture water demand and concrete strength both at normal hardening and after steaming has been obtained. Compatibility and efficiency indicators of different SP types are calculated for concrete mixtures based on low-clinker SPC and for various curing conditions. 

## 2. Materials and Methods Used in the Research

The present research was performed for concrete based on cement with the following composition: clinker—11.2%, slag—81.8%, phosphogypsum—7.0% (SO_3_—4.2%). The cement-specific surface area was 4534 cm^2^/g and the standard cement strength was 48 MPa. Locally available granulated blast furnace slag attributed to the main slags (M_o_ = 1.09) [1], Dickerhoff clinker cement (C_3_S = 57.1%, C_2_S = 21.27%, C_3_A = 6.87%, and C_4_AF = 12.19%), which according to its mineralogical composition can be attributed to typical medium aluminate clinkers produced by cement plants of Ukraine. Phosphogypsum dihydrate (FG) was used as a sulfate component of low-clinker slag Portland cement (LCSPC). Chemical composition of the materials is given in Table 2. Naphthalene–formaldehyde and polycarboxylate superplasticizers are currently the most common, however, they significantly increase the concrete mix cost. The cheapest plasticizers are lignosulfonates, and modification of lignosulfonates by various known methods makes it possible to bring their effect closer to that of superplasticizers. Therefore, plasticizing admixtures used narthium lignosulfonate (LS), Polyplast (SP-1)—a condensation product of naphthalene sulfonic acids and formaldehyde, NF and Sika ViscoCrete 225—polycarboxylate ether (P). The admixtures were added to the concrete mix in a dry state. Granite crushed stone with a maximum grain size of 20 mm and sand with fineness modulus M*_f_* = 1.9 were used as aggregates for concrete.

The research was performed using mathematical experiment planning. With this aim, a three-level, two-factor plan was implemented [24]. The experiments’ planning conditions are given in Table 3. The concrete mixture slump at all points of the plan was 70–130 mm. The concrete mix water demand was determined by finding the necessary workability [25]. Cubes with a size of 100 × 100 × 100 mm were prepared and tested according to [26] to obtain the compressive strength at 7 and 28 days. In addition, concrete specimens were subjected to steaming at 80 °C. The rate of heating and cooling was 30 °C per hour. The isothermal exposure duration was 6 h. Steamed concrete specimens were tested to obtain the strength immediately after steaming, as well as 28 days after steaming. For each strength test, three specimens were used. For each experimental point (Table 4), a series of 36 specimens was tested. The total number of specimens is 396. 

The matrix of experiment planning and the results of testing are presented in Table 4. 

Based on the experimental results and statistical processing of data (Table 4), the mathematical experimental–statistical models of the investigated properties of concrete with additives were obtained. Such models take into account the influence of the investigated factors (Table 3).

Since most of the dependencies used for the solution of structural and technological problems in concrete technology have a second-order polynomial form [24], it was decided to apply an experimental plan that allows obtaining just such models. The mathematical model of second order, in our case, has the following form:

y = b_0_ + b_1_*X*_1_ + b_2_*X*_2_ + b_11_*X*_1_^2^ + b_12_*X*_2_^2^ + b_12_*X*_1_*X*_2_, (3)

where y is the output parameter (concrete properties);

b_0_, b_1_, b_2_, b_22_, b_11_, b_12_ are regression coefficients (constants);

*X*_1_, *X*_2_ are the investigated factors (variables).

The data obtained by testing the specimens was processed to obtain equations the form of Equation (3). In this way, mathematical models of water consumption of concrete mixture and compressive strength of concrete were obtained (Table 5). Equations in this table have a general form of Equation (3). In these equations, coefficient b_0_ corresponds to the arithmetic mean value of the investigated property, obtained in the experiment; b_1_, b_2_ display the linear influence of the investigated factors (*X*_1_ and *X*_2_, Table 3); b_11_, b_22_ display the non-linear influence of the investigated factors; b_12_ displays the combined influence of factors *X*_1_ and *X*_2_.

## 3. Analysis of the Results

**Figure 1:**
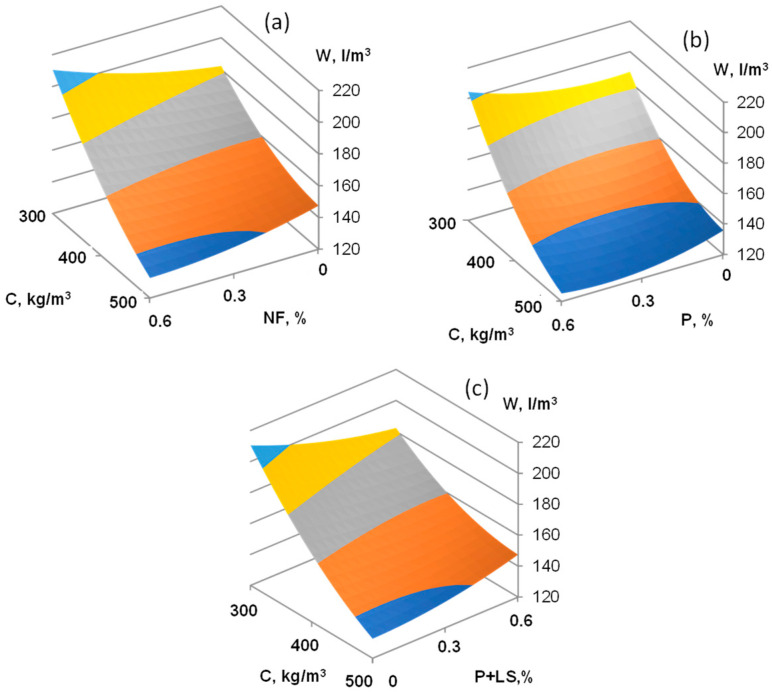
The combined effect of cement consumption and various types of superplasticizers (NF (**a**), P (**b**), and P+LS (**c**)) on water demand of concrete based on low-clinker slag Portland cement.

**Figure 2:**
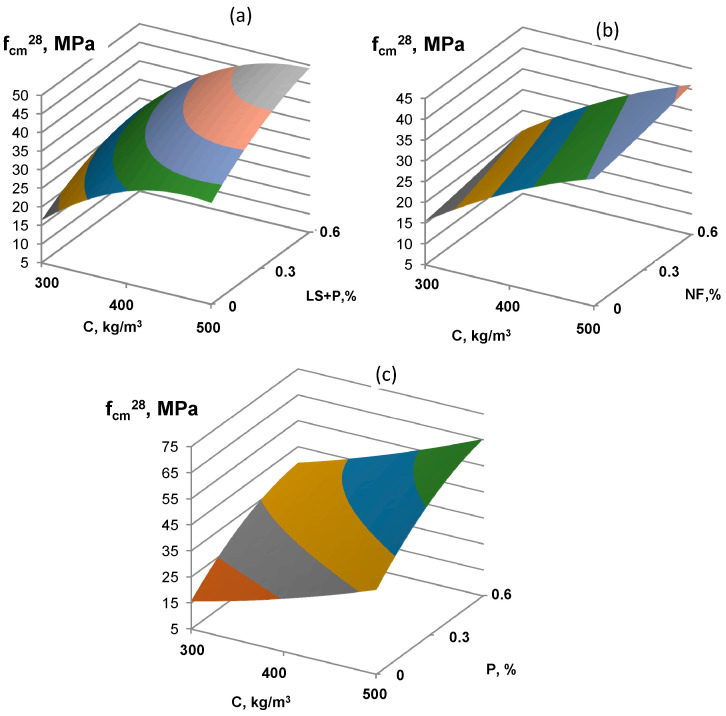
The combined effect of cement consumption and various types of superplasticizers (NF (**a**), P (**b**), and P+LS (**c**)) on low-clinker slag Portland cement concrete strength at 28 days.

**Figure 3:**
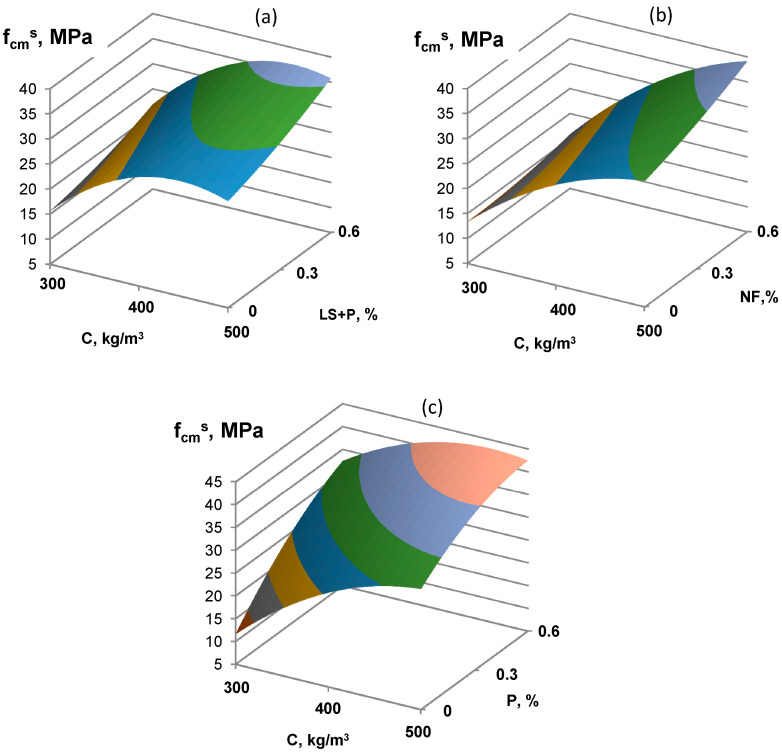
The combined effect of cement consumption and various types of superplasticizers (NF (**a**), P (**b**), and P+LS (**c**)) on low-clinker slag Portland cement concrete strength after steaming.

**Figure 4:**
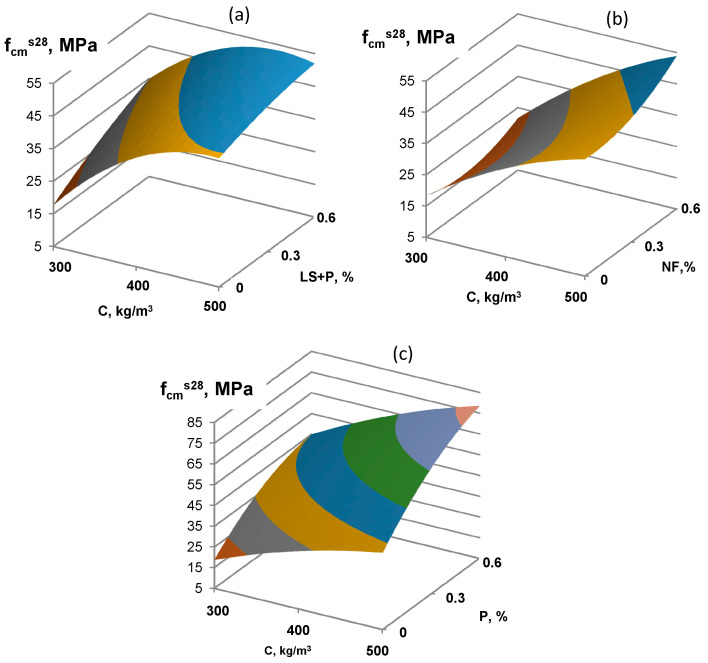
The combined effect of cement consumption and various types of superplasticizers (NF (**a**), P (**b**), and P+LS (**c**)) on low-clinker slag Portland cement concrete strength after TMP and 28 days of normal hardening.

Graphical dependences that illustrate the influence of technological factors on the concrete mixture water demand and compressive strength of concrete after steaming and 28 days of normal hardening after steaming are shown in Figure 1, Figure 2, Figure 3 and Figure 4. The obtained results show that the plasticizer content has the most significant effect on the concrete mixture water demand (Figure 1). When the SP content is increased to 0.6% by the cement weight, the concrete mixture water demand is reduced by 20% for NF and by 35% for P and P+LS plasticizers. It was also found that at SP content of 0.3% by cement weight, the highest water-reducing effect is observed for using P-type SP, worse—for P and LS-type SP in amounts of 1:1, and worst when using NF-type SP.

The concrete strength at 7 and 28 days increases with an increase in binder content and SP content, which leads to a corresponding decrease in *W/C*. Increasing the content of P admixture to the value of 0.6% by cement weight allows maximal *W/C* reduction and, accordingly, doubles the magnitude of the concrete strength. At the same time, the concrete strength is about 40 MPa at 7 days and more than 60 MPa at 28 days. When using P and LS SP it is about 30 MPa at 7 days and more than 45 MPa at 28 days; for NF SP it is 30 MPa and 40 MPa at 7 and 28 days, respectively.

The relatively high strength of concrete based on low-clinker slag Portland cement can be explained by high binder reactivity, which has an increased grinding fineness, contributing to a higher activating effect of the binder cement and sulfate components on the blast furnace slag. Achieving low water-cement ratio values due to the use of plasticizing admixtures contributes to cement hydration in “compressed conditions”, which leads to faster formation of a supersaturated solution, with such a supersaturation degree, at which hydrate neoplasms and hardening structure formation occurs most rapidly [1].

An even more significant effect of using finely ground low-clinker slag Portland cement is observed during heat-moist processing (Figure 3 and Figure 4). A characteristic feature of concrete on LCSPC is a more intense increase in strength after steaming. If the specimens’ strength 4 h after steaming was 45 MPa, then at 28 days, the strength was 80 MPa for concrete with P-type SP, 35 MPa and 50 MPa for P and LS-type SP, 40 MPa, and 50 MPa for NF-type SP.

To evaluate the effectiveness and compatibility of various superplasticizers with an LCSPC according to the mathematical models (Equations (4)–(18)), a comparison of the admixtures’ water-reducing effect and the relative increase in strength at 7 and 28 days was carried out. 

**Figure 5:**
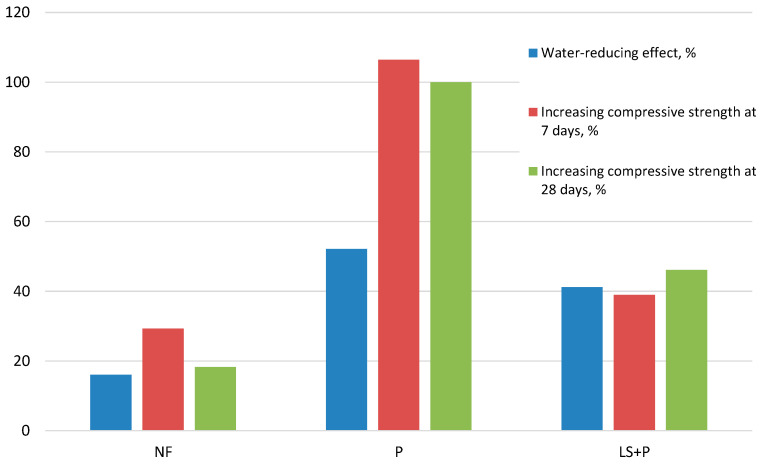
Comparative diagram of water-reducing effect and effect of increasing compressive strength at 7 and 28 days caused by SP.

A graphic comparison of these indicators is shown in Figure 5. The effect of naphthalene–formaldehyde polymer NF on LCSPC-based concrete caused an average water demand decrease of 13.9%; the corresponding strength increased at 7 and 28 days were 29.3% and 18.3%, respectively. For the complex P and LS admixture, the water-reducing effect was 29.2%, and the increase in strength was 38.9% and 46.1% at 7 and 28 days, correspondingly. Pure polycarboxylate polymer (P) caused a decrease in concrete mixture water demand by 34.3%, while the compressive strength increased more significantly: at 7 days by 106%, and at 28 days—by 100%. Considering that the water-reducing effect of admixtures indicates a possible change in the concrete water–cement ratio (i.e., the cement stone porosity), then the obtained data indicate that the effect of superplasticizers is based not only on reducing porosity but also on a positive effect on cement hydration. This is especially noticeable for polycarboxylate superplasticizers, for which the typical water-reducing effect is close to 35%.

According to theoretical concepts [27], the high strength of concrete can be ensured by reducing *W/C* to the maximum possible values with a simultaneous increase in cement hydration degree. According to Powers [28], the compressive strength of cement stone specimens hardened under normal conditions corresponds to the following equation:

(19) $$f_{C.S}=AX^{n},$$

where 

*A* is a constant characterizing the cement gel strength (*A* ≈ 240 MPa), 

*n* is a coefficient determined by cement characteristics (*n* = 2.6–3),

*X* is a structural criterion.

Structural criterion *X* in Equation (19) characterizes the cement hydration products concentration in the space available for these substances and is proportional to the relative cement stone density:

(20) $$X=\frac{K_{H}V_{PC}\alpha }{V_{PC}\alpha +W/C}\approx \frac{0.47\alpha }{0.319\alpha +W/C},$$

where 

*K_H_* = 2.09–2.2 is the hydration products’ (gel) volume growth coefficient; 

*V_PC_* is the specific volume of cement (*V_PC_* = 1/*ρ*_C_ = 0.319 cm^3^/g is a value inverse to the cement density (*ρ*_C_); 

*α* is part of the cement that has undergone hydration (hydration degree). 

Known experimental data also shows a higher increase in the strength of cement paste with low *W/C* values and a relatively small increase in the hydration degree [1]. For example, at *W/C* = 0.2 with an increase in α from 0.1 to 0.2, the cement stone compressive strength increases from 30 to 55 MPa, and already at *W/C* = 0.3—only from 15 to 25 MPa [29].

Table 6 shows the concrete strength change ratio ∆*f_cm_* and the SP water-reducing effect under different hardening and duration conditions of LCSPC concrete. This criterion uniquely characterizes the efficiency and compatibility of the investigated superplasticizer types and cement.

According to the experimental data (Table 6), the maximum values of the proposed *K_c_* criterion for polycarboxylate SP additive were 1.82–3.11, the NF-type SP was slightly less effective (*K_c_* = 1.32–2.12). Naphthalene–formaldehyde-type SP was quite effective when used with LCSPC, especially at an early age (on 7th day *K_c_* = 2.12). SP, consisting of polycarboxylate and lignosulfonate, showed high efficiency at 28 days of normal hardening (*K_c_* = 1.58). It is because polycarboxylate superplasticizers have a significantly higher water-reducing effect due to the steric liquefaction mechanism. This allowed the significant reduction of the concrete mixture water demand and *W/C*, improving the concrete strength and a number of other properties.

## 4. Conclusions

Experimental and statistical models for water demand and compressive strength of LCSPC concrete with a clinker consumption of 12% at 7 and 28 days were obtained for cases of normal hardening and steaming and using superplasticizers based on naphthalene formaldehyde, polycarboxylate, and lignosulfonate polymers.

It was found that polycarboxylate SP is characterized by the maximum water-reducing effect (27 ... 52%) of LCSPC concrete, the complex polycarboxylate and lignosulfonate SP is somewhat less effective (19–41%), and the lowest effect (10–17%) was found for naphthalene formaldehyde SP. At the same time, polycarboxylate SP demonstrated an increase in concrete strength by two times (more than 60 MPa at 28 days). For polycarboxylate and lignosulfonate superplasticizers, the strength was more than 45 MPa, and for naphthalene–sulformaldehyde—about 40 MPa.

The use of the investigated SP types and LCSPC allows for achieving a significant increase in concrete strength, even after heat and moisture processing. The effectiveness of various SP types, allowing to achieve concrete strength from 50 MPa to 80 MPa has been found.

A criterion for evaluating the compatibility and effectiveness of the investigated superplasticizers with cements, considering the SP water-reducing effect and the corresponding relative change in strength (*K_c_*), is proposed. The values of the criterion indicate cement and SP compatibility and indicate the additional effect that SP have on structure formation and, as a result, on concrete strength.

## Figures and Tables

**Table 1:** Classification of superplasticizers.

Notation	Composition	Effect
NF	Based on sulfonated naphthalene–formaldehyde polycondensates	Electrostatic
MF	Based on sulfonated melamine–formaldehyde polycondensates	Electrostatic
LST	Based on sugar-free lignosulfonates	Electrostatic
P	Based on polycarboxylates and polyacrylates	Steric

**Table 2:** Chemical composition of the initial materials.

Type of Materials	Content of Oxides by Weight, %							
	SiO_2_	Al_2_O_3_	Fe_2_O_3_	CaO	MgO	SO_3_	MnO	P_2_O_5_
Granulated blast furnace slag	39.52	6.49	0.12	47.13	3.10	1.74	1.15	-
Phosphogypsum	-	0.36	0.15	38.4	0.003	59.7	-	0.67
Clinker	22.47	5.26	4.07	66.18	0.64	0.46	0.29	-

**Table 3:** Experiments planning conditions.

No.	Factors		Variation Levels			Variation Interval
	Natural	Coded	−1	0	1	
1	Binder consumption, kg/m^3^ (C)	*X*_1_	300	400	500	100
2	Content of plasticizing admixtures, %: NF, P, P+LS (1:1)	*X*_2_	0	0.3	0.6	0.3

**Table 4:** Experiments results.

No	Planning Matrix		Water Concumption, L			Compressive Strength at 7 Days, MPa			Compressive Strength at 28 Days, MPa			Compressive Strength Immediately after Steaming, MPa			Compressive Strength after Steaming and 28 Days of Normal Curing, MPa		
	*X*_1_	*X*_2_	NF	P	LS+P	NF	P	LS+P	NF	P	LS+P	NF	P	LS+P	NF	P	LS+P
1	1	1	168	125	132	30.5	41.4	28.6	41.0	65.3	49.0	39.1	42.4	35.7	53.8	78.0	52.0
2	−1	1	201	204	211	237	25.8	21.4	35.6	37.5	32.2	30.2	31.3	26.3	42.3	41.7	44.4
3	1	−1	162	136	148	14.3	23.8	18.4	20.0	38.9	26.9	15.9	32.4	21.9	21.7	44.5	35.0
4	−1	−1	182	187	182	12.3	13.0	10.4	15.4	15.9	16.3	13.4	11.5	15.7	18.4	18.4	17.8
5	0	1	184	153	164	26.0	32.2	27.1	38.1	56.0	43.0	34.0	39.4	30.2	44.5	65.6	49.3
6	0	−1	172	150	157	12.2	17.0	16.5	17.5	32.0	23.9	14.0	24.5	18.0	16.5	37.2	27.5
7	1	0	164	125	137	19.4	25.6	27.1	32.3	50.8	43.4	30.8	41.5	34.6	40.6	63.9	50.8
8	−1	0	190	190	193	15.0	12.4	19.5	27.3	25.4	29.7	25.1	25.5	26.8	33.2	32.7	38.4
9	0	0	177	144	157	16.1	17.6	25.4	29.6	42.7	38.9	27.3	36.0	29.9	33.4	54.0	45.7
10	0	0	178	145	158	16.5	17.3	25.1	29.6	42.1	38.5	27.1	36.3	30.2	33.3	54.2	45.8
11	0	0	177	145	156	16.0	17.6	25.6	29.4	43.0	38.5	27.3	36.1	29.7	33.6	53.8	45.2

**Table 5:** Experimental and statistical models of water demand and strength of concrete on low-clinker slag Portland cement.

Plasticizer Type	Experimental—Statistical Models	
Concrete mixture water demand		
NF	W = 177.3 + 6.168*X*_1_ − 13.169*X*_2_ + 0.936*X*_1_^2^ − 0.064*X*_2_^2^ − 3.5*X*_1_*X*_2_	(4)
P	W = 145.3 + 1.667*X*_1_ − 32.507*X*_2_ + 5.894*X*_1_^2^ + 11.894*X*_2_^2^ − 7.0*X*_1_*X*_2_	(5)
P+LS	W = 157.1 + 3.334*X*_1_ − 28.172*X*_2_ + 3.285*X*_1_^2^ + 7.785*X*_2_^2^ − 10.75*X*_1_*X*_2_	(6)
Compressive strength at 7 days		
NF	*f_cm_*^7^ = 16.118 + 6.985*X*_1_ + 2.217*X*_2_ + 3.014*X*_1_^2^ + 1.114*X*_2_^2^ + 1.2*X*_1_*X*_2_	(7)
P	*f_cm_*^7^ = 17.656 + 7.552*X*_1_ + 6.651*X*_2_ + 6.969*X*_1_^2^ + 1.369*X*_2_^2^ + 1.2*X*_1_*X*_2_	(8)
P+LS	*f_cm_*^7^ = 25.372 + 5.251*X*_1_ + 3.817*X*_2_ − 3.618*X*_1_^2^ − 2.118*X*_2_^2^ − 0.2*X*_1_*X*_2_	(9)
Compressive strength at 28 days		
NF	*f_cm_*^28^ = 29.612 + 10.252*X*_1_ + 2.517*X*_2_ − 1.764*X*_1_^2^ + 0.236*X*_2_^2^ + 0.2*X*_1_*X*_2_	(10)
P	*f_cm_*^28^ = 42.659 + 12.036*X*_1_ + 12.74*X*_2_ + 1.275*X*_1_^2^ − 4.625*X*_2_^2^ + 1.2*X*_1_*X*_2_	(11)
P+LS	*f_cm_*^28^ = 38.988 + 9.52*X*_1_ + 6.851*X*_2_ − 5.451*X*_1_^2^ − 2.351*X*_2_^2^ + 1.575*X*_1_*X*_2_	(12)
Compressive strength immediately after steaming		
NF	*f_cm_*^s^ = 27.31 + 10.04*X*_1_ + 2.851*X*_2_ − 3.31*X*_1_^2^ + 0.64*X*_2_^2^ + 1.625*X*_1_*X*_2_	(13)
P	*f_cm_*^s^ = 36.076 + 7.451*X*_1_ + 7.968*X*_2_ − 4.123*X*_1_^2^ − 2.473*X*_2_^2^ − 2.45*X*_1_*X*_2_	(14)
P+LS	*f_cm_*^s^ = 29.921 + 6.118*X*_1_ + 3.934*X*_2_ − 5.789*X*_1_^2^ + 0.761*X*_2_^2^ + 0.8*X*_1_*X*_2_	(15)
Compressive strength after steaming and 28 days of normal hardening		
NF	*f_cm_*^s28^ = 33.44 + 14.086*X*_1_ + 3.70*X*_2_ − 2.92*X*_1_^2^ + 3.53*X*_2_^2^ + 2.05*X*_1_*X*_2_	(16)
P	*f_cm_*^s28^ = 54.04 + 14.24*X*_1_ + 15.64*X*_2_ − 2.576*X*_1_^2^ − 5.74*X*_2_^2^ + 2.55*X*_1_*X*_2_	(17)
P+LS	*f_cm_*^s28^ = 45.73 + 10.969*X*_1_ + 6.168*X*_2_ − 7.346*X*_1_^2^ − 1.146*X*_2_^2^ − 2.4*X*_1_*X*_2_	(18)

**Table 6:** Values of *K_c_* = ∆*f_cm_*/WRE under different curing conditions and duration of LCSPC concrete.

Curing Conditions	Type of Superplasticizer		
	NF	P	P+LS
Normal hardening, 7 days	2.12	3.11	1.34
Normal hardening, 28 days	1.32	2.92	1.58
Steaming	1.64	1.82	1.00

Note: cement consumption—400 kg/m^3^, SP content—0.6% by cement weight.

## Data Availability

Not applicable.
